# Comprehensive analysis of TP53 and SPOP mutations and their impact on survival in metastatic prostate cancer

**DOI:** 10.3389/fonc.2022.957404

**Published:** 2022-08-31

**Authors:** Jie Zhou, Yiming Lai, Shengmeng Peng, Chen Tang, Yongming Chen, Lingfeng Li, Hai Huang, Zhenghui Guo

**Affiliations:** ^1^ Department of Urology, Sun Yat-sen Memorial Hospital, Sun Yat-sen University, Guangzhou, China; ^2^ Guangdong Provincial Clinical Research Center for Urological Diseases, Sun Yat-Sen Memorial Hospital, Sun Yat-Sen University, Guangzhou, China

**Keywords:** metastatic prostate cancer, TP53, SPOP, prognosis, biomarkers, mutation

## Abstract

**Background:**

Although TP53 and SPOP are frequently mutated in metastatic prostate cancer (PCa), their prognostic value is ambiguous, and large sample studies are lacking, especially when they co-occur with other genetic alterations.

**Methods:**

Genomic data and patients’ clinical characteristics in PCa were downloaded from the cBioPortal database. We extensively analyzed other gene alterations in different mutation status of TP53 and SPOP. We further subdivided TP53 and SPOP mutation into subgroups based on different mutation status, and then evaluated the prognostic value. Two classification systems for TP53 survival analysis were used.

**Results:**

A total of 2,172 patients with PCa were analyzed in our study, of which 1,799 were metastatic PCa patients. The mutual exclusivity analysis showed that TP53 and SPOP mutation has a strong mutual exclusion (p<0.001). In multivariable analysis, truncating TP53 mutations (HR=1.773, 95%CI:1.403-2.239, p<0.001) and other TP53 mutations(HR=1.555, 95%CI:1.267-1.908, p<0.001) were independent negative prognostic markers in metastatic PCa, whereas SPOP mutations(HR=0.592, 95%CI:0.427-0.819, p<0.001) were an independent prognostic factor for better prognosis. Mutations in TP53 were significantly associated with wild-type status for SPOP and CDK12, structural variants/fusions for TMPRSS2 and ERG, AR amplification and PTEN deletion (p<0.001). And truncating TP53 mutations have higher AR amplification rates than other TP53 mutations (p=0.022). Consistently, truncating TP53 mutations had a worse prognosis than other TP53 mutations (p<0.05). Then Kaplan-Meier survival curve showed that Co-occurring TP53 mutations in AR amplification or PTEN deletion tumors significantly reduced survival (p<0.05). Furthermore, those with SPOP-mutant tumors with co-occurring TP53 truncating mutations had shorter overall survival than those with SPOP-mutant tumors with wild-type or other TP53 mutations.

**Conclusions:**

This study found that TP53 and SPOP mutations were mutually exclusive and both were independent prognostic markers for metastatic PCa. Genomic alteration and survival analysis revealed that TP53 and SPOP mutations represented distinct molecular subtypes. Our data suggest that molecular stratification on the basis of TP53 and SPOP mutation status should be implemented for metastatic PCa to optimize and modify clinical decision-making.

## Introduction

Prostate cancer (PCa) is the second most common cancer in men. More than 1.2 million new cases are diagnosed each year, accounting for 7% of all newly diagnosed cancers in men worldwide. In addition, more than 350,000 prostate cancer-related deaths occur globally each year, making it one of the leading causes of cancer-related deaths in men (1).

TP53 gene is the most frequently mutated gene in human cancers. This gene encodes a tumor suppressor protein p53, which is a master regulator of a variety of physiological and pathological processes including DNA repair, cell cycle, senescence and cell death (2). Like many other malignant tumors, TP53 is one of the most frequently mutated genes in human PCa and is enriched in later stages (3). TP53 mutations are observed in 10–20% of cases of localized PCa, and the frequency of mutations increases significantly or even exceeds 50% in advanced cases (4). The mutated p53 protein elicits a variety of dysregulations, ranging from total loss-of-function to gain-of-function mutations. Each mutation has different features. Functional consequences of TP53 mutations may depend on the specific mutation or the type of mutations.

Nowadays, with the advancement of gene sequencing technology, somatic gene mutation analysis is more and more widely used in clinical practice (5). Different mutation status can be used to identify different molecular subtypes and thus predict prognosis and guide treatment. Since TP53 is widely mutated in human cancers, numerous studies have tried to answer the question of the impact of TP53 mutations on patient survival (6–8). For PCa, there are fewer relevant findings and a lack of large sample studies on the effect of TP53 mutations on prostate cancer survival (3, 9). A recently published study on the prognostic impact of TP53 or DNA damage repair gene mutations concluded that TP53 mutations were an independent risk factor for PSA failure or PSA persistence and can be used to define a subgroup of patients in primary PCa (9). Moreover, TP53 mutations were reported to be associated with metastasis and castration resistance (10, 11). However, although TP53 mutations are common in metastatic PCa, it is unclear whether patients with TP53 mutations have a reduced overall survival time compared to patients without TP53 mutations, and it is also unclear whether this effect is dependent on other genetic alterations.

To study the prognostic impact of TP53 mutations, different TP53 classification systems have been used in squamous-cell carcinoma of the head and neck, lung cancer and metastatic colorectal cancer (6, 8, 12). No matter how, given the diversity of effects caused by different types of TP53 mutations, the dichotomy of TP53 status into “wild-type” and “mutant” underestimates the complexity and leads to low-resolution results.

SPOP, an E3 ubiquitin ligase adaptor, is also one of the most frequently mutated genes in PCa (13, 14). By participating in the ubiquitinated proteasome system (UPS), SPOP plays an important role in regulating the levels and activities of a variety of proteins as well as in regulating the cell cycle, gene expression, response to oxidative stress, cell survival, cell proliferation and autophagy (15, 16). Due to the diversity of regulatory pathways and the variety of tumor types involved, SPOP has recently received increasing attention (17, 18). Abnormalities in SPOP function can disrupt downstream biological processes and promote tumorigenesis (19). For PCa, SPOP mutations occurs in 5-15% of patients (4). Although the sample size is relatively small, recently published studies have shown that SPOP mutations may be associated with a better prognosis (20). Larger sample size studies are urgently needed to confirm the effect of SPOP mutations on overall survival time, especially in lethal metastatic PCa.

As mentioned above, both TP53 and SPOP are the most frequently mutated genes in PCa. However, despite the fact that they are both located on chromosome 17 and play important roles in oxidative stress, cell survival and cell death, direct analysis of the association of TP53 and SPOP mutations in metastatic PCa is still lacking. In liquid biopsies, a study showed that TP53 was a better predictor of drug sensitivity to abiraterone or enzalutamide than androgen receptor biomarkers in metastatic castration-resistant prostate cancer (mCRPC) (10). Another study also noted that men with PCa with compound tumor suppressor gene mutations had poorer outcomes and may benefit from intensified treatment (11). SPOP, like TP53, also functions as a tumor suppressor in PCa (18). Nevertheless, the literature reported that SPOP mutations in PCa, unlike TP53 mutations, could instead enhance sensitivity to androgen deprivation therapy (ADT) (20). Large studies investigating the effects of TP53 and SPOP mutations on patient survival could help to translate the molecular findings into clinical practice.

In this study, we performed a comprehensive analysis of TP53 and SPOP mutations. In a large cohort of patients with metastatic PCa, we compared SPOP mutation status and different TP53 classification systems to establish an easy-to-use prognostic classification with an emphasis on therapeutic decisions. We also extensively analyzed other gene alterations in different mutation status of TP53 and SPOP. Furthermore, for the first time, we evaluated the relationship between the most frequent TP53 mutations and SPOP mutations in PCa and systematically analyzed the effect of different TP53 mutations on OS in SPOP-mutated patients.

## Materials and methods

### Gene mutations and clinical characteristics in the cBioPortal database

Information regarding TP53 and SPOP mutations and patients’ clinical characteristics in PCa was downloaded from the cBioPortal Database, an open-access database that is publicly available at: http://www.cbioportal.org. We chose MSK MetTropism (MSK, Cell 2021) as our data source, which is a pan-cancer study and has been published in the journal (21). The majority of participants in this study were metastatic cancer patients. The total number of patients was 25,775, of which the number of PCa patients was 2172. One tumor sample was taken from each patient. A total of 2172 tumor samples were profiled using the MSK-IMPACT targeted sequencing platform, including 1,312 primary PCa and 860 metastases. Among primary PCa, 373 were from non-metastatic PCa patients and 939 were from patients with metastatic disease (21). Therefore, tumor samples from a total of 1799 patients with metastatic PCa were included in our survival analysis.

In cBioPortal, users can input specific cases of interest by selecting “User-Defined Case List” (22). This allows us to study gene mutations in patients with different metastatic status. The gene of interest, “TP53:MUT” and/or “SPOP:MUT”, were entered in the input box. Gene mutations as well as clinical data were downloaded from the cBioPortal website after submitting the query. Data was merged according to the unique patient ID, such as “P-0001202”. For patients with metastatic PCa, we downloaded 1799 pieces of mutation data and clinical data. After merging, excluding 5 patients with missing survival data, the remaining 1794 patients contained mutation type of TP53 and SPOP, survival time and the status of patient (living or deceased).

This study has the following characteristics: ①all patients were from the same center (Memorial Sloan Kettering Cancer Center), which ensures uniformity in diagnostic criteria, treatment regimens, surgical methods and medications; ②all samples were sequenced between 2013-11-18 and 2020-01-06 (6.1y), which is a concentrated period of time, and the diagnostic and treatment regimens received by the patients will not change significantly during the period; ③All patients were clearly diagnosed with prostate adenocarcinoma, excluding neuroendocrine cancer/intraductal carcinoma;④Patients included in the survival analysis were all mPCa patients with multiple metastatic sites (mean 2.967) and metastatic lesions (mean 3.824); ⑤Patients were grouped to compare prognosis, and the grouping was based on the results of the gene mutation test without any subjective factors, which also avoided some bias.

We divided TP53 and SPOP mutation into different groups based on the mutation site in different exons. The lollipop of TP53 and SPOP mutation was from cBioPortal website.

No statements of approval or informed consent were required for our study as we obtained data from an open-access database.

### TP53 mutation classification

We evaluated somatic TP53 mutations and survival in patients with metastatic PCa. Two classification systems for TP53 survival analysis were used. One is to classify TP53 status into wild type and mutant type. The other regards the “technical” type of mutation, separating frameshift, nonsense and splice mutations (termed “TP53truncating”), from all other mutations, including missense, synonymous, and in-frame mutations (termed “TP53others”) (6). For SPOP mutation analysis, we concentrated on mutation status with no further subdivision.

### Statistics

Statistical analyses were performed by Statistical Package for the Social Sciences (IBM SPSS Statistics 23) and GraphPad Prism 8. The distribution of time to event was analyzed using Kaplan-Meier statistics and compared between groups by log-rank test. Association of qualitative variables was tested by chi-square or Fisher’s exact test, depending on distributional assumptions. Univariable analyses or multivariable Cox proportional hazard model were used to analyze associations between mutations and patient survival. Overall survival (OS) data was obtained from the cBioPortal website directly. Kaplan-Meier survival curves and forest plots were plotted using GraphPad Prism 8. Statistical significance was set at 0.05, and all p values are two-sided.

## Results

### Mutation rate of TP53 and distribution of different exon

**Figure 1:**
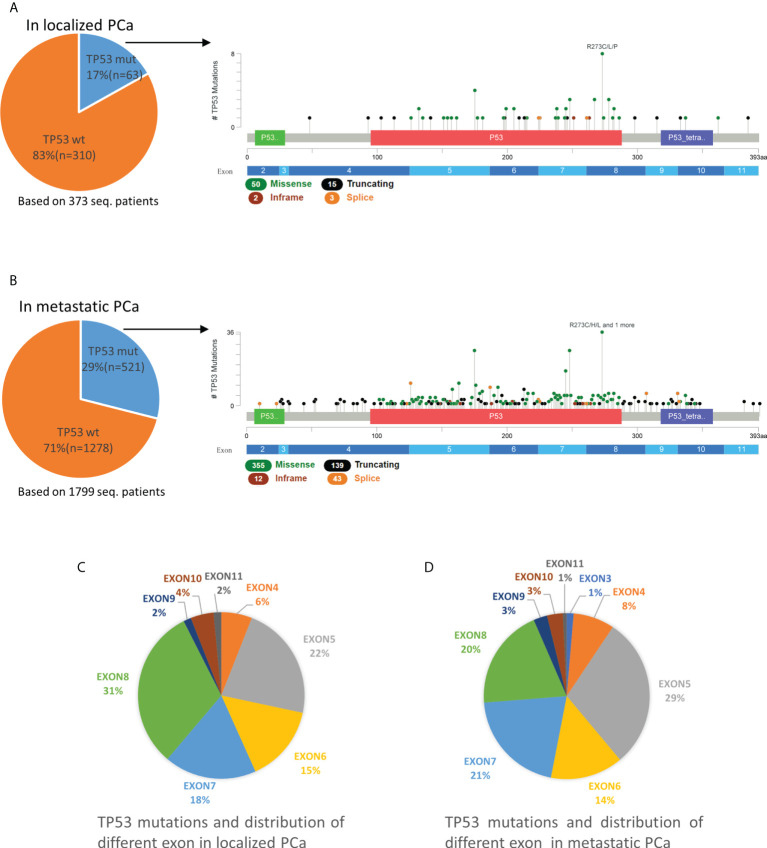
Mutation mapping for TP53 in localized PCa **(A)** and metastatic PCa **(B)**. Distribution of different TP53 mutation site in localized PCa **(C)** and metastatic PCa **(D)**. aa, amino acid; seq., sequencing; TP53wt, TP53 wild type; TP53mut, TP53 mutation.

In 373 non-metastatic (localized) PCa patients, the mutation rate of TP53 was 17% (63/373). Exons 5-8 were the most frequent mutation sites in TP53, accounting for 22%, 15%, 18% and 31%, respectively (**Figure 1**). In 1799 metastatic PCa patients, the mutation rate was 29% (521/1799), which was significantly higher than in localized PCa (p<0.001). The mutation rates of exons 5-8 were 29%, 14%, 21% and 20%, respectively. Unlike localized PCa, which has the highest rate of exon 8 mutations, the rate of exon 5 mutations was highest in metastatic PCa. Exons 2, 3, 4, 9, 10, and 11 were less frequently mutated in all PCa patients (**Figure 1**).

For TP53, most mutations were missense mutations, followed by truncating (including splice) and inframe mutations (**Figure 1**). In localized PCa patients, TP53truncating accounts for 27% (17/63) of patients with TP53 mutations and 26% (18/70) of total TP53 mutations. Multiple mutations occurred in 11% (7/63) of patients. In metastatic PCa patients, TP53truncating accounts for 34% (179/521) of patients with TP53 mutations and for 33% (182/549) of total TP53 mutations, which was not statistically different compared to patients with localized PCa (p>0.05). Multiple mutations occurred in 5% (28/521) patients.

### Mutation rate of SPOP and distribution of different exon

**Figure 2:**
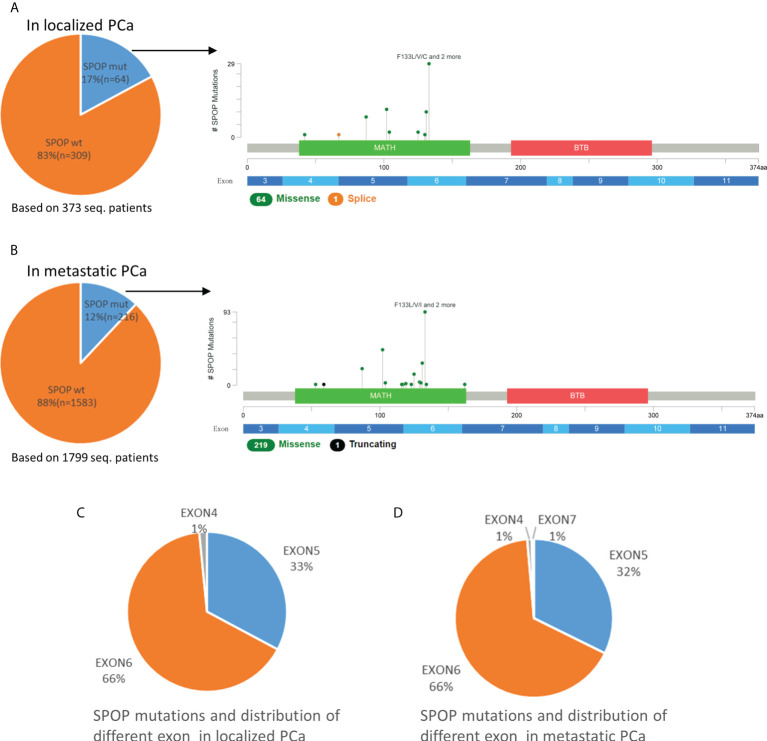
Mutation mapping for SPOP in localized PCa **(A)** and metastatic PCa **(B)**. Distribution of different SPOP mutation site in localized PCa **(C)** and metastatic PCa **(D)**. aa, amino acid; seq., sequencing; SPOPwt, SPOP wild type; SPOPmut, SPOP mutation.

In 373 localized PCa patients, the mutation rate of SPOP was 17% (64/373). However, in 1799 cases of metastatic PCa, the SPOP mutation rate dropped to 12% (216/1799), with a statistically significant difference (p<0.01). Whether in localized or metastatic PCa, the SPOP mutation types are almost always missense mutations, followed by the very rare truncating or splice mutations (**Figure 2**). Missense mutations were mostly clustered in codons 133, 102, 131, and 87. Exons 5-6 were the most frequent mutation sites and did not differ in the proportion of localized and metastatic PCa (**Figure 2**). SPOP Multiple mutations were rare. In metastatic PCa patients, multiple mutations account for only 2% (4/216) of patients with SPOP mutations. Overall, SPOP mutations in metastatic PCa had a decreased mutation rate compared with localized PCa, while mutation types and mutation sites did not differ between the two groups.

#### Correlation of TP53 mutation and SPOP mutation

**Figure 3:**
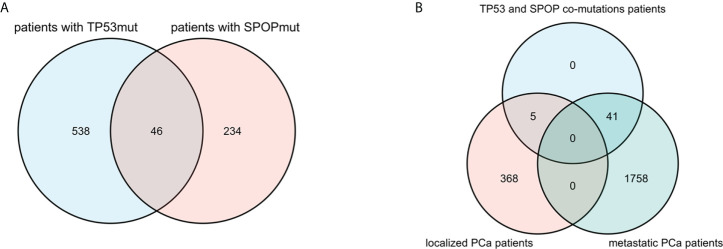
Venn diagram showed that there were 46 patients with TP53 and SPOP co-mutation **(A)**. Distribution of co-mutation patients in localized PCa and metastatic PCa **(B)**.

Among all 2172 PCa patients, there were 584 patients with TP53 mutation and 280 patients with SPOP mutation, but only 46 patients with co-mutation of both (**Figure 3A**). In cBioPortal, the mutual exclusivity analysis (22) showed that TP53 mutation and SPOP mutation have the strongest tendency toward mutual exclusivity, and the relationship was statistically significant (p<0.001). In 1799 metastatic PCa patients, there were 521 patients with TP53 mutation and 216 patients with SPOP mutation, and 41 patients with concurrent mutations. The mutual exclusivity analysis also showed the same result as above. Furthermore, we also found that co-mutations were not associated with PCa metastasis (p=0.252) (**Figure 3B**).

### Significant genomic alterations in metastatic PCa patients with TP53 or SPOP mutations

Mutations in TP53 were significantly associated with wild-type status for SPOP and CDK12, structural variants/fusions for TMPRSS2 and ERG, AR amplification and PTEN deletion (**Table 1**). Patients with TP53 mutations were further subdivided into TP53truncating and TP53other. Compared to other TP53 mutations patients, truncating TP53 mutations were only significantly associated with AR amplification (p=0.022). In TP53 truncating and TP53 other mutations patients, the rate of AR amplification was 32.4% and 23.1%, respectively (**Table 1**). Although truncating TP53 mutations appeared to be associated with wild-type status for SPOP, there was no statistical difference compared to TP53 other mutations (p=0.081).

**Table 1:** Significant genomic alterations between TP53wt and TP53mut tumors.

Variants	Total, No. 1799	Patients with TP53wt, No.(%) 1278	Patients with TP53mut, No.(%) 521	χ2	P value	Patients with TP53 truncating mutations, No.(%) 179	Patients with TP53 “other mutations” No.(%) 342	χ2	P value
SPOP				11.882	0.001			3.037	0.081
wild type	1583 (88.0)	1103 (86.3)	480 (92.1)			170 (95.0)	310 (90.6)		
mutant	216 (12.0)	175 (13.7)	41 (7.9)			9 (5.0)	32 (9.4)		
CDK12				28.649	<0.001			0.227	0.696
wild type	1690 (93.9)	1176 (92.0)	514 (98.7)			176 (98.3)	338 (98.8)		
mutant	109 (6.1)	102 (8.0)	7 (1.3)			3 (1.7)	4 (1.2)		
AR (amplification)				75.159	<0.001			5.247	0.022
yes	268 (14.9)	131 (10.3)	137 (26.3)			58 (32.4)	79 (23.1)		
no	1531 (85.1)	1147 (89.7)	384 (73.7)			121 (67.6)	263 (76.9)		
TMPRSS2 (structural variants / fusions)				38.314	<0.001			0.073	0.787
yes	559 (31.1)	342 (26.8)	217 (41.7)			76 (42.5)	141 (41.2)		
no	1240 (68.9)	936 (73.2)	304 (58.3)			103 (57.5)	201 (58.8)		
ERG (structural variants / fusions)				29.064	<0.001			0.081	0.776
yes	471 (26.2)	289 (22.6)	182 (34.9)			64 (35.8)	118 (34.5)		
no	1328 (73.8)	989 (77.4)	339 (65.1)			115 (64.2)	224 (65.5)		
PTEN (deletion)				31.898	<0.001			0.031	0.859
yes	270 (15.0)	153 (12.0)	117 (22.5)			41 (22.9)	76 (22.2)		
no	1529 (85.0)	1125 (88.0)	404 (77.5)			138 (77.1)	266 (77.8)		

TP53wt, TP53 wild type; TP53mut, TP53 mutation.

Mutations in SPOP were significantly associated with wild-type status for TP53, mutant APC, less structural variant/fusion for TMPRSS2 and ERG, less AR amplification and PTEN deletion (**Table 2**). All these findings were consistent with the results of the mutual exclusivity analysis described above.

**Table 2:** Significant genomic alterations between SPOPwt and SPOPmut tumors.

Variants	Total, No. 1799	Patients with SPOPwt, No.(%) 1583	Patients with SPOPmut, No.(%) 216	χ^2^	P value
TP53				11.882	0.001
wild type	1278 (71.0)	1103 (69.7)	175 (81.0)		
mutant	521 (29.0)	480 (30.3)	41 (19.0)		
TP53					
wild type	1278 (71.0)	1103 (69.7)	175 (81.0)	13.966	0.001
other	342 (19.0)	310 (19.6)	32 (14.8)		
truncating	179 (10.0)	170 (10.7)	9 (4.2)		
APC				51.24	<0.001
wild type	1665 (92.6)	1491 (94.2)	174 (80.6)		
mutant	134 (7.4)	92 (5.8)	42 (19.4)		
TMPRSS2 (structural variants/fusions)				97.864	<0.001
yes	559 (31.1)	555 (35.1)	4 (1.9)		
no	1240 (68.9)	1028 (64.9)	212 (98.1)		
ERG (structural variants/fusions)				78.07	<0.001
yes	471 (26.2)	468 (29.6)	3 (1.4)		
no	1328 (73.8)	1115 (70.4)	213 (98.6)		
AR (amplification)				5.185	0.023
yes	268 (14.9)	247 (15.6)	21 (9.7)		
no	1531 (85.1)	1336 (84.4)	195 (90.3)		
PTEN (deletion)				20.729	<0.001
yes	270 (15.0)	260 (16.4)	10 (4.6)		
no	1529 (85.0)	1323 (83.6)	206 (95.4)		

SPOPwt, SPOP wild type; SPOPmut, SPOP mutation.

#### Impact of TP53 mutation on the prognosis of metastatic PCa

**Figure 4:**
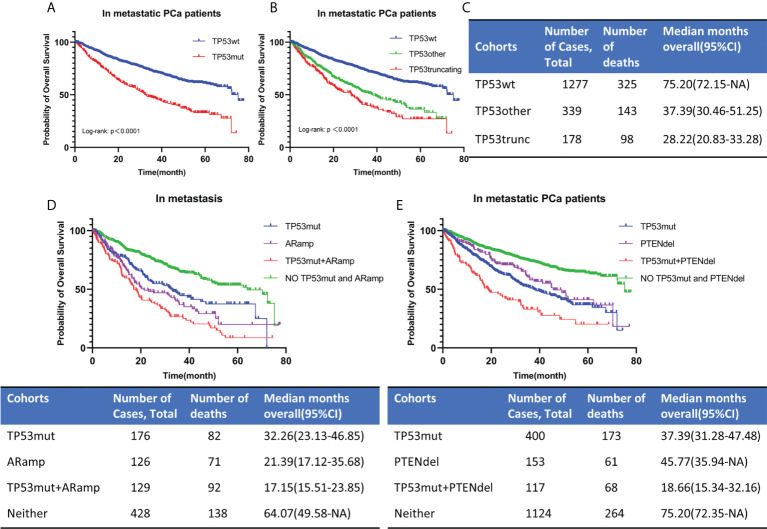
OS for metastatic PCa according to TP53 mutation status **(A, B)**. TP53 classification by means of mutational type (see the Methods section). The estimated median OS for different TP53 mutation status **(C)**. OS for metastatic PCa when AR amplification or PTEN deletion co-occurred with TP53 mutation **(D, E)**. TP53wt, TP53 wild type; TP53mut, TP53 mutation; ARamp, AR amplification; PTENdel, PTEN deletion; OS, overall survival; CI, confidence interval; NA, not applicable.

OS was analyzed in 1794 patients with metastatic PCa. The follow-up period ranged from 0 to 77.7 months, with a median follow-up time for censored patients of 22.56 months. Among the 1794 patients, there were 517 patients with TP53 mutation and 1277 patients with TP53 wild-type. Firstly, we estimated the prognostic impact of TP53 by mutation or not. Kaplan-Meier survival curve indicated that TP53 mutation was associated with a poor prognosis (**Figure 4A**). The estimated median OS for TP53 wild-type cohort (TP53wt) and TP53 mutation cohort (TP53mut) was 75.2 months and 32.26 months, respectively, (p<0.0001). Then, in another classification, we divided TP53 status into 3 cohorts, namely TP53wt, TP53other and TP53truncating (**Figure 4B**). Among the 517 TP53mut patients, there were 339 patients with TP53other and 178 patients with TP53truncating. Kaplan-Meier survival curve showed that TP53truncating has a worse prognosis compared to TP53other (**Figure 4B**). The estimated median OS for TP53other cohort and TP53truncating cohort was 37.39 months and 28.22 months, respectively (**Figure 4C**). The difference was statistically significant (p<0.05).

Since exon 5 and exon 8 have different mutation rates in localized and metastatic PCa, to explore whether their mutations have an impact on the survival of patients with metastatic PCa, we further subdivided TP53mut into 3 cohorts with different mutated exons, namely exon 5, exon 8 and exon other. Excluding patients with missing or unclear exon site information, 495 patients were included in our study, and the total number of mutated exons was 522. Multiple exon mutations were present in 26 patients and were included in the exon other cohort. The number of patients included in exon 5, exon 8 and exon other cohorts were 141, 92 and 262, respectively. Kaplan-Meier survival curve showed that exon 5 and 8 mutations have no significant effect on OS (p=0.3876) (**Additional File: Figure 1**).

As mentioned above, mutations in TP53 were significantly associated with AR amplification and PTEN deletion (**Table 1**). In our study, AR amplification was largely seen in metastasis samples. Then we divided 860 patients into 4 cohorts according to TP53 mutation and AR amplification (**Figure 4D**). Patients without TP53 mutation and AR amplification had the best prognosis, while those with co-occurrence of TP53 mutation and AR amplification had the worst prognosis. The estimated median OS for AR amplification cohort and TP53 mutation and AR amplification co-occurrence cohort was 21.39 months and 17.15 months, respectively. The difference was statistically significant (p=0.0447). According to TP53 mutation and PTEN deletion, we divided 1794 metastatic PCa patients into 4 cohorts (**Figure 4E**). Patients with co-occurring TP53 mutations and PTEN deletions still had the worst prognosis, with a predicted median OS time of 18.66 months. The difference was statistically significant compared to patients with only TP53 mutation or PTEN deletion (P<0.0001).

In summary, TP53 mutations were associated with poor prognosis in metastatic PCa. TP53 truncating mutations had a worse prognosis than TP53 other mutations, whereas different mutated exon was not associated with prognosis. When AR amplification or PTEN deletion co-occurred with TP53 mutation in metastatic PCa, the prognosis of patients was significantly worse than that of patients with a single change. However, we should also be aware that the prognostic impact of AR amplification was correlated with previous therapies and castration-resistance.

#### Impact of SPOP mutation on the prognosis of metastatic PCa

**Figure 5:**
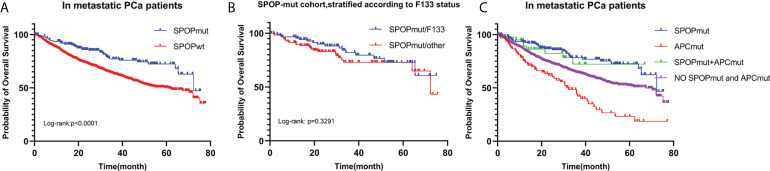
OS for metastatic PCa according to SPOP mutation status **(A)**. Patients with SPOP mutations were further stratified according to F133 mutation site **(B)**. OS for metastatic PCa in different SPOP/APC mutation groups **(C)**. SPOPwt, SPOP wild type; SPOPmut, SPOP mutation; APCmut, APC mutation; OS, overall survival.

216 SPOPmut and 1578 SPOPwt patients with metastatic PCa were included in the analysis. Kaplan-Meier survival curve showed that SPOPmut was associated with a better prognosis (**Figure 5A**). The estimated median OS for SPOPwt cohort and SPOPmut cohort was 60.68 months (95% CI: 49.58-72.15months) and 72.35 months (95% CI: 65.35-NA months), respectively. The difference was statistically significant (p<0.0001). SPOP mutations were almost always missense mutations, and the F133 mutation was the most common mutation site. In view of the role of the SPOP F133 mutation (23), we then divided the 216 SPOP mutated patients into SPOPmut/F133 cohort (92) and SPOPmut/other cohort (124) according to whether F133 was mutated or not (**Figure 5B**). Kaplan-Meier survival curve showed no statistical difference between the two cohorts (p=0.3291).

As mentioned above, Mutations in SPOP were significantly associated with APC mutations. According to SPOP mutations and APC mutations status, we divided 1794 metastatic PCa patients into 4 cohorts (**Figure 5C**). Patients with only APC mutations had the worst prognosis, with a predicted median OS time of 32.26 months. The differences were statistically significant compared to the other three cohorts (P<0.0001). In contrast, the prognosis of patients in the SPOP and APC co-mutation cohort was not significantly different from that of patients in the SPOP mutation cohort (P=0.720).

Taken together, these results showed that SPOP mutations were associated with a good prognosis, but mutation sites were not prognostic related. And SPOP mutations could lead to survival benefits in the context of APC mutations.

### Prognostic value of TP53 and SPOP mutation in metastatic PCa

**Figure 6:**
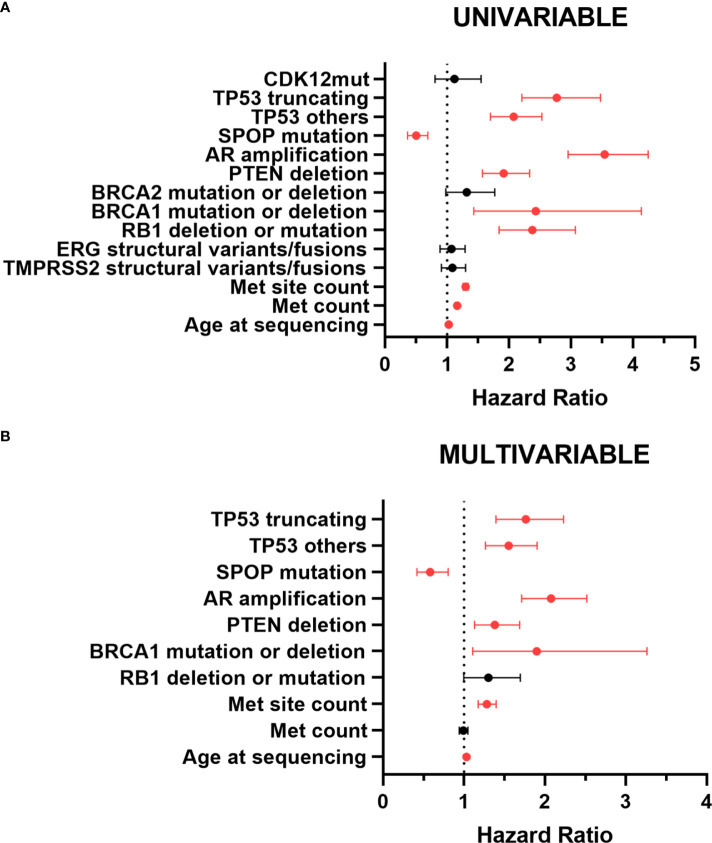
Forest plot for prognostic factors in patients with metastatic PCa by univariable **(A)** and multivariable **(B)** analyses. The forest plot reveals the HRs and 95% confidence intervals of the prognostic factors. Red color indicates significant p values. Met, metastatic; mut, mutation; HR, hazard ratio.

For univariable analysis, we included the following variables: age at sequencing, metastatic count, metastatic site count, mutant status of TP53, SPOP and CDK12, AR amplification, PTEN deletion, RB1 deletion or mutation, BRCA1 mutation or deletion, BRCA2 mutation or deletion, and structural variant/fusion of TMPRSS2 and ERG (**Additional File: Table 1**). Univariable analysis revealed a significantly higher risk of death for metastatic PCa patients with high age at sequencing, more metastatic count and metastatic site count, TP53 truncating mutations, TP53 other mutations, RB1 deletion or mutation, BRCA1 mutation or deletion, AR amplification and PTEN deletion. However, Patients with mutated SPOP had a significantly lower risk of death (**Figure 6A**). And CDK12 mutations, BRCA2 mutation or deletion, and structural variant/fusion of TMPRSS2 and ERG had no effect on survival.

In multivariable analysis, meaningful variables described above remained significant except metastatic count and RB1 deletion or mutation. TP53 mutations were still significantly associated with poor prognosis (**Figure 6B**). Compared with TP53 wild-type, the hazard ratio (HR) for death events in patients with TP53 other mutations was 1.555 (95% CI,1.267-1.908; p<0.001). TP53 truncating mutations patients had a worse prognosis. The HR for death events was 1.773 (95% CI, 1.403-2.239; p<0.001). Unlike TP53 mutations, SPOP mutations were associated with a better prognosis. The HR for death events was 0.592 (95% CI, 0.427-0.819; p=0.002). Taken overall, TP53 mutations, especially TP53 truncating mutations, were significantly associated with shorter survival, thus representing an independent negative prognostic factor for metastatic PCa, whereas SPOP mutations were an independent prognostic factor for better prognosis.

#### Impact of TP53 and SPOP co-mutations on the prognosis of metastatic PCa

TP53 mutation and SPOP mutation were the most common mutations in PCa. Although they were mutually exclusive, there were still a small number of patients with co- mutations. TP53 mutation predicted poor prognosis, while SPOP mutation showed the opposite. To the best of our knowledge from reading the literature, no studies on the prognosis of patients with TP53 and SPOP co-mutations have been reported.

**Figure 7:**
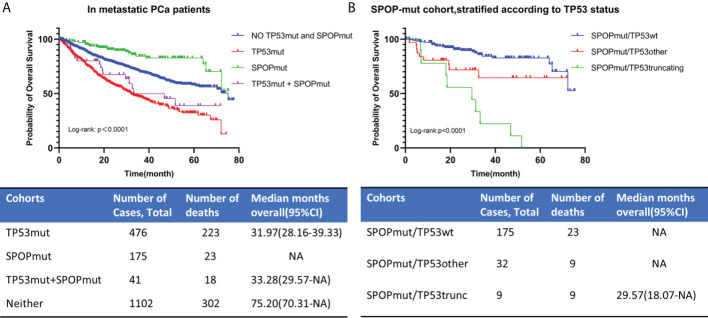
OS for metastatic PCa in different TP53/SPOP mutation groups **(A)**. Patients with SPOP mutations were further stratified according TP53 mutation status **(B)**. TP53wt, TP53 wild type; TP53mut, TP53 mutation; SPOPmut, SPOP mutation; OS, overall survival; CI, confidence interval.

1794 patients with metastatic PCa were divided into no TP53 and SPOP mutation cohort, TP53 mutation cohort, SPOP mutation cohort, and TP53 and SPOP co-mutation cohort according to their TP53 and SPOP mutation status (**Figure 7A**). Kaplan-Meier survival curve showed no significant difference in prognosis between TP53mut cohort and co-mutation cohort. The estimated median OS for TP53mut cohort and co-mutation cohort was 31.97 (28.16-39.33) months and 33.28 (29.57-NA) months, respectively, (p=0.236). However, Kaplan-Meier survival curve showed that the prognosis differed significantly between SPOPmut cohort and co-mutation cohort (p<0.0001). To further explore the effect of different TP53 mutation types on SPOP mutations, we divided metastatic PCa patients with SPOP mutation into three cohorts according to TP53 mutation status, namely SPOPmut/TP53wt cohort, SPOPmut/TP53other cohort and SPOPmut/TP53truncating cohort (**Figure 7B**). Statistical differences were found between all three cohorts. Patients in the SPOPmut/TP53truncating cohort had the worst prognosis. All these results suggested that TP53 mutation played a greater role in patients with co-mutations and could mask the prognostic impact of SPOP mutation.

## Discussion

TP53 and SPOP mutations are the most common mutations in PCa. However, the prognostic role of TP53 and SPOP mutations in PCa has not been fully elucidated (20), which can be partly attributed to the difficulty of long follow-up, the difficulty in obtaining large sample sizes, unstandardized study approaches and the fact that some sequencing panels do not include TP53 and SPOP (9). Due to the good prognosis of localized PCa and the lack of large sample sequencing data, in this study, we focused on the effect of TP53 and SPOP mutations on OS in lethal metastatic PCa.

Here, a total of 2172 patients with PCa, including 1799 patients with metastatic PCa, were analyzed in this study. Compared with localized PCa, TP53 mutation rate was elevated in metastatic PCa, while SPOP mutation rate was slightly decreased. Metastatic PCa patients with tumors harboring TP53 truncating mutations or TP53 other mutations had significantly shorter OS compared with those with TP53wt. And the prognosis of TP53 truncating mutations was worse than that of other mutations in TP53, while the different mutated exons had no significant effect on the prognosis. These results point to different oncogenic effects of truncating versus other and missense mutations. Unlike TP53, SPOP mutations are a marker of a better prognosis in metastatic PCa. However, when SPOP-mutant tumors harbor additional TP53 truncating or other mutations, patients lived significantly shorter lives.

For TP53, defining three subgroups on the basis of mutation types was crucial to identify patients in the subgroup with shorter OS. In metastatic PCa, truncating rather than missense TP53 mutations lead to shortened survival. This means that loss of TP53 expression, a typical consequence of truncating TP53 mutations (24), worsens patients’ conditions. Previous literature has reported that the majority of TP53 mutations are missense mutations compared to other tumor suppressor genes that are mainly altered by truncating mutations (25, 26). As we have shown, the same is true in metastatic PCa, where the major types of TP53 mutations are missense mutations. Missense mutations of the TP53 gene result in a mutant p53 protein with a single amino acid substitution, which leads to a deleterious gain-of-function. A possible explanation for the difference in survival of truncating TP53 mutations or missense TP53 mutations is that the mutated p53 protein may still execute some of its tumor-suppressive functions in metastatic PCa (6). However, we still need to keep in mind that in metastatic PCa even patients with TP53 missense mutations have a significantly worse prognosis than patients with TP53wt. It is therefore plausible to believe that total loss-of-function effects of p53 play a larger role in metastatic PCa.

Multiple TP53 classification systems have been applied to human cancer analysis. In advanced non-small-cell lung cancer, TP53 mutation is also a negative prognostic factor and different mutated exon has different prognostic value (7). In our study, there were no similar results found. In some studies, the large group of TP53 missense mutations has been further stratified (27). No matter how, a dichotomous analysis regarding TP53 mutation status is insufficient, leads to low-resolution results, and should be abandoned.

Most previous studies on SPOP mutations did not distinguish between localized and metastatic PCa, and the sample sizes were relatively small (20, 28). Our study is the largest study up to date examining the prognostic impacts of SPOP mutations on patients with metastatic PCa. This observation deserves special attention because mutant SPOP renders tumor cells susceptible to androgen deprivation therapy and target SPOP might become therapeutic strategies in the future (18, 28–31). The possible reason why SPOP mutated PCa patients are sensitive to abiraterone is that mutant SPOP can promote 17betaHSD4 protein degradation to drive androgenesis (32). In addition, SPOP mutations are associated with a high degree of genomic instability and deficiency in homologous recombination repair of DNA (33). With our work, we found that SPOP mutation status was an important independent prognostic marker in metastatic PCa. This was consistent with the results reported in the relevant literature. Therefore, we recommend adding SPOP mutation testing to the treatment and diagnostic protocols of metastatic PCa.

For the first time, we analyzed the relationship between TP53 mutations and SPOP mutations using large sample sequencing data and systematically analyzed the effect of different TP53 mutation status on OS in SPOP-mutated metastatic PCa patients. To our surprise, our findings showed that the most frequently mutated TP53 and SPOP in PCa have a strong mutual exclusion. This was consistent with the results of the prognostic analysis and suggested that they represented two distinct subtypes of PCa. However, due to the high mutation rate, even in the presence of mutual exclusivity, some patients with metastatic PCa still have co-mutations in both TP53 and SPOP, as shown by our findings. Although patients with SPOP mutations had a better prognosis than patients without SPOP mutations, co-occurring TP53 mutations in SPOP-mutated tumors significantly reduced survival. These findings were consistent with the previously reported results of significantly shorter time-to-castration-resistance in SPOP-mutant patients with concurrent TP53 mutations (20).

Furthermore, significant genomic alterations in metastatic PCa patients with different TP53 or SPOP status were evaluated. Patients with TP53 mutations had a significantly higher chance of AR amplification, PTEN deletion, and TMPRSS2-ERG fusion. Co-occurring TP53 mutations in tumors significantly reduced survival owing to poor response to ADT treatment (34, 35). In the mouse prostate, loss of TP53 can enhance the AR-mediated oncogenic transformation and tumor development (36). Higher rates of AR amplification in truncating TP53 mutations were also associated with worse treatment response and prognosis. In contrast, lower rates of AR amplification in patients with SPOP mutations were associated with better response to ADT treatment and prognosis. The relationship between mutant SPOP and APC has been recently reported (20). And for the first time, our work suggested that in metastatic PCa, unlike TP53 and SPOP co-mutations, co-occurring SPOP mutations in APC-mutated tumors lead to a survival benefit. Further research is needed to investigate the exact interactions of mutant SPOP and APC.

Recently, efforts have been made to integrate molecular markers more into the clinical management of PCa (29, 37, 38). Patients with localized stage disease do not routinely undergo molecular analysis, and current recommendations are to limit next-generation sequencing (NGS) to mCRPC patients. Germline and somatic mutation testing of the HRR gene are now used clinically to predict the efficacy of PAPR inhibitors (39). In contrast, many other mutations that have clinical value in metastatic PCa are still not recommended for testing (40). This leads to low-resolution risk stratification. High-risk patients who could benefit from different treatment modalities may be missed (11). In our daily practice, we used NGS in metastatic PCa patients to test the mutations of HRR genes, but the test for TP53 and SPOP is not routinely performed till now. To improve the prognosis of patients with metastatic PCa, more precise molecular subtype stratification to guide treatment is necessary. As we have shown, TP53 and/or SPOP mutations occurred in nearly half of patients with metastatic PCa, and they both predicted ADT treatment response and prognosis. Therefore it can be assumed that our comprehensive analysis could help to optimize the treatment of patients with metastatic PCa.

Limitations of our study are due to the nature of the analyzed data and biases of this analytical approach. As reported in other literature (7), the present study is a pure clinical study based on database. Our results were not validated in other cohorts because the data in the TCGA database mainly included patients with localized PCa and a limited number of patients with metastatic PCa. The mechanisms underlying the differences in prognosis among different mutation status still need further investigation, especially when patients have gene co-mutations.

In summary, the large sample size of our cohort provides unparalleled statistical power to the question of the prognostic properties of TP53 and SPOP mutations in metastatic PCa. Our data suggest that genetic testing should include TP53 and SPOP and should be broadened to include all metastatic prostate cancers. Identification of worse prognostic groups on the basis of TP53 and/or SPOP mutation status can help to modify and optimize clinical treatment decisions in metastatic PCa.

## Data availability statement

The original contributions presented in the study are included in the article/**Supplementary Material**. Further inquiries can be directed to the corresponding authors.

## Ethics statement

The studies involving human participants were reviewed and approved by Human Oncology and Pathogenesis Program, Memorial Sloan Kettering Cancer Center, New York, NY, USA. The patients/participants provided their written informed consent to participate in this study.

## Author contributions

JZ, ZG, HH, YL, and SP conceived and designed the study, participated in the collection of data and data analysis, and drafted the manuscript. CT assisted in the design of this research and project development. YC and LL analyzed the data and reviewed the article. All authors contributed to the article and approved the submitted version.

## Funding

This work was supported by the National Natural Science Foundation of China (No: 81772733, 81972384), Guangdong scientific research projects (No: 2021A1515010223) to ZG. And also supported by the National Natural Science Foundation for Young Scientists of China (No: 81802527) and Beijing Bethune Charitable Foundation (No: mnzl202026) to YL. In addition, this work was supported by the National Natural Science Foundation of China (No: 81974395, No: 82173036); Guangdong Basic and Applied Basic Research Foundation (No: 2019A1515011437); International Science and technology cooperation project plan of Guangdong Province (No: 2021A0505030085); Sun Yat-Sen University Clinical Research 5010 Program (No: 2019005); Sun Yat-Sen Clinical Research Cultivating Program (No: 201702); Guangdong Province Key Laboratory of Malignant Tumor Epigenetics and Gene Regulation (No:2020B1212060018OF006); Guangdong Provincial Clinical Research Center for Urological Diseases (2020B1111170006) Beijing Bethune Charitable Foundation (mnzl202001) Guangzhou Science and Technology Key R&D Project (202206010117) to Hai Huang.

## Conflict of interest

The authors declare that the research was conducted in the absence of any commercial or financial relationships that could be construed as a potential conflict of interest.

## Publisher’s note

All claims expressed in this article are solely those of the authors and do not necessarily represent those of their affiliated organizations, or those of the publisher, the editors and the reviewers. Any product that may be evaluated in this article, or claim that may be made by its manufacturer, is not guaranteed or endorsed by the publisher.
