# Supplementary material for: Comprehensive analysis of TP53 and SPOP mutations and their impact on survival in metastatic prostate cancer
Source: Front Oncol. 2022 Aug 31;12:957404. doi: 10.3389/fonc.2022.957404 (PMC9471084; doi:10.3389/fonc.2022.957404)
Supplement: Supplementary file 1 [file DataSheet_1.docx]

**ADDITIONAL FILES**


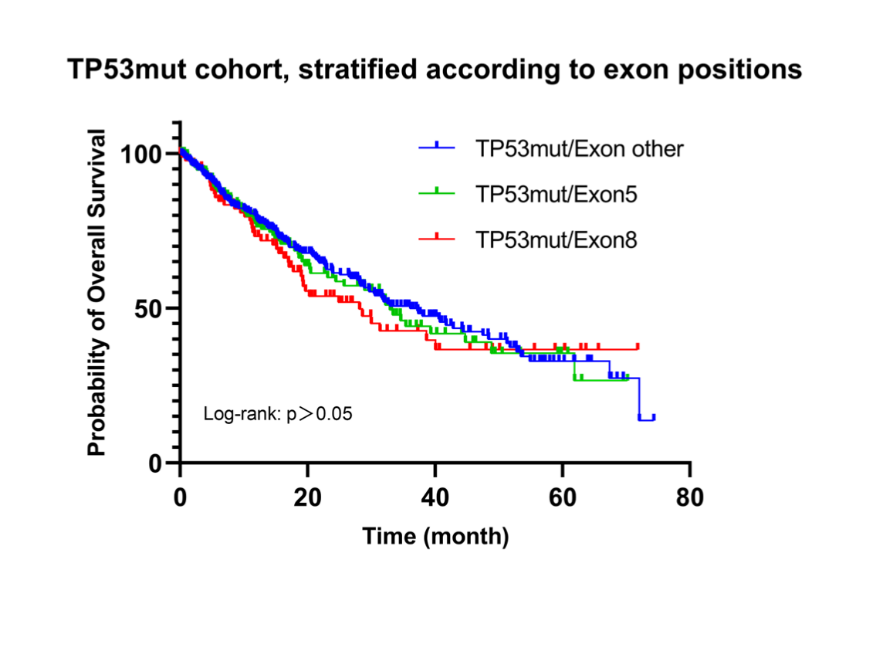


**FIGURE 1.** Overall survival in TP53 mutated patients subdivided into exon 5, exon 8 and exon other mutation.

**TABLE 1.** Prognostic factors by univariable and multivariable analyses

|  | Univariate Cox | | |  | Munivariate Cox | | |
| --- | --- | --- | --- | --- | --- | --- | --- |
|  | HR | 95% CI | P value |  | HR | 95% CI | P value |
| Age at seq | 1.028 | 1.019-1.038 | 0.000 |  | 1.032 | 1.022-1.042 | 0.000 |
| Met site count | 1.298 | 1.259-1.339 | 0.000 |  | 1.279 | 1.172-1.396 | 0.000 |
| Met count | 1.162 | 1.141-1.183 | 0.000 |  | 0.993 | 0.940-1.048 | 0.789 |
| ERG(structural variants / fusions) | 1.070 | 0.887-1.292 | 0.479 |  |  |  |  |
| TMPRSS2(structural variants / fusions) | 1.086 | 0.909-1.298 | 0.363 |  |  |  |  |
| CDK12mut | 1.118 | 0.808-1.549 | 0.500 |  |  |  |  |
| PTEN deletion | 1.915 | 1.572-2.333 | 0.000 |  | 1.355 | 1.107-1.660 | 0.003 |
| AR amplification | 3.541 | 2.954-4.245 | 0.000 |  | 2.034 | 1.679-2.464 | 0.000 |
| SPOPmut | 0.500 | 0.363-0.690 | 0.000 |  | 0.592 | 0.427-0.819 | 0.002 |
| TP53other | 2.075 | 1.701-2.533 | 0.000 |  | 1.555 | 1.267-1.908 | 0.000 |
| TP53trunc  RB1 deletion or mutation  BRCA1 mutation or deletion  BRCA2 mutation or deletion | 2.769  2.377  2.433  1.318 | 2.206-3.476  1.840-3.070  1.431-4.136  0.980-1.772 | 0.000  0.000  0.001  0.067 |  | 1.773  1.301  1.900 | 1.403-2.239  0.999-1.696  1.107-3.260 | 0.000  0.051  1.900 |
